# ^18^F-FDG PET/CT and Whole-Body Bone Scan Findings in Gorlin–Goltz Syndrome

**DOI:** 10.3390/diagnostics13132239

**Published:** 2023-06-30

**Authors:** Miju Cheon, Jang Yoo, Kyu-Bok Kang

**Affiliations:** 1 Department of Nuclear Medicine, Veterans Health Service Medical Center, Seoul 05368, Republic of Korea; 2 Department of Orthopaedic Surgery, Veterans Health Service Medical Center, Seoul 05368, Republic of Korea

**Keywords:** Gorlin–Goltz syndrome, Gorlin’s syndrome, basal cell nevus syndrome, ^18^F-FDG, PET/CT, whole-body bone scan

## Abstract

Gorlin–Goltz syndrome (basal cell nevus syndromes) is an uncommon, autosomal dominant inherited disorder characterized by developing basal cell carcinomas from a young age. Other distinct clinical features include keratocystic odontogenic tumors, dyskeratotic palmar and plantar pitting, and skeletal abnormalities. Clinicopathological findings of the syndrome are very diverse, and many symptoms manifest during a certain period of life. We present the compelling whole-body bone scan and ^18^F-FDG PET/CT findings in a 32-year-old man with odontogenic keratocyst, early-onset basal cell carcinoma, multiple ectopic calcifications in extremities, calcified falx cerebri, spinal scoliosis, macrocephaly, and ocular hypertelorism.

**Figure 1:**
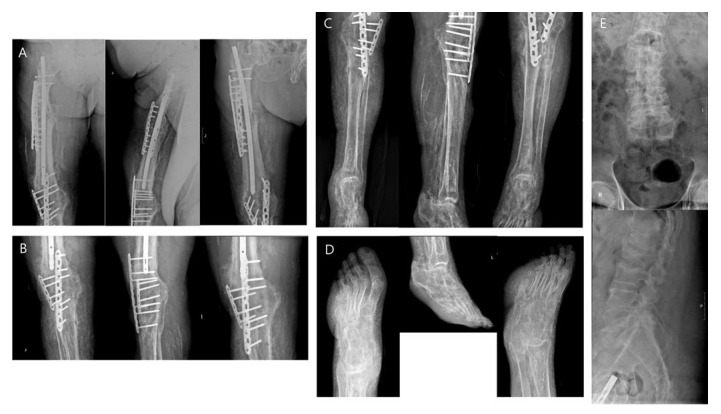
A 32-year-old male was referred to the Department of Nuclear Medicine to evaluate long-standing left leg pain. About 7 years ago, he had left femur shaft, patella, and tibia fractures in a car accident and had a history of undergoing open reduction and internal fixation. Moreover, about 5 years ago, a nonunion of the left proximal tibia fracture caused knee floating, and it was managed with knee fusion using a Charnley clamp. A year ago, he suffered multiple injuries in a car accident, including a left distal femur fracture. The fractures were managed with open reduction and internal fixation with a bone graft. He complained of persistent pain in his left lower leg despite multiple surgeries. Over the years, he has taken many plain radiographs, including of the left lower extremity. These studies demonstrated diffuse osteopenia, pseudocyst lesions, a moth-eaten appearance to the trabecular pattern, several radiolucencies, a permeative pattern, an old fracture, and multiple soft tissue calcifications (Figure 1). Plain radiographs of the left femur (**A**), left knee (**B**), left tibia (**C**), left foot (**D**), and lumbar spines (**E**). Plain radiographs of the lower limb were of the anterior view, lateral view, and oblique view in order from left to right, and in the case of the L-spine, the anterior view and lateral view were arranged in order from the top. In all plain radiographs of the left lower extremity, numerous abnormal calcified opacities of various sizes were observed in soft tissue. Additionally, it showed diffuse osteopenia, several radiolucencies, or a permeative pattern. The left first toe showed a pseudocystic lytic lesion on its medial side. Deformities were also in the left fourth and fifth toes. The L-spine radiograph revealed spondylolytic spondylolisthesis of L5 on S1, degenerative disk disease of the lumbar spine, and compression fracture at the L1 and L2 spines.

**Figure 2:**
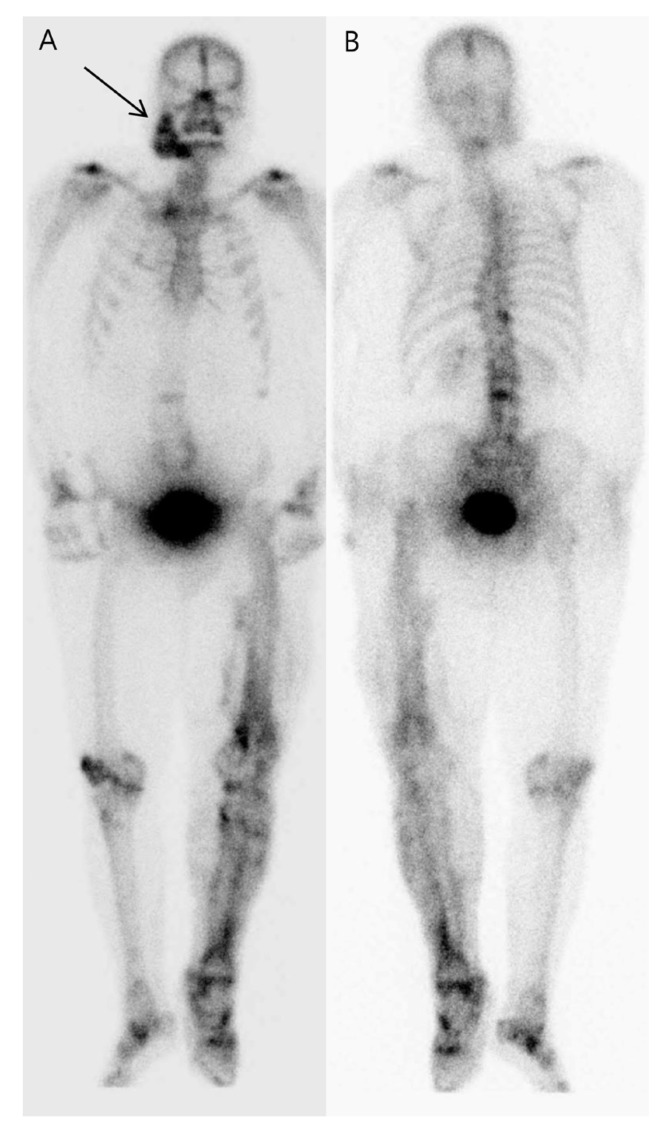
The whole-body bone scan was obtained 4 h after the intravenous injection of 740 MBq of technetium-99m methylene diphosphonate (MDP), using a gamma camera with a gantry motion of 10 cm/min and a matrix size of 256 × 1024. It showed marked increased radiotracer uptake in the central photopenic area in the right mandible. Multiple abnormal soft tissue radioactive uptake lesions were observed at the level from the left mid-to-distal thigh to the left foot, and the radioactivity uptake was slightly increased in the lower thoracic spine and the L3 spine. Based on these findings, fibrous dysplasia or an odontogenic tumor was first considered for the right mandible lesion. The possibility of heterotopic ossifications was considered for the uptake lesions in the soft tissue of the left lower extremity, which were consistent with the distribution of abnormal calcified opacities seen in the conventional plain radiograph. In the case of spinal lesions, when referring to X-ray findings, it was judged to be caused by degenerative disc disease and compression fracture due to scoliosis, although it was early onset compared to the patient’s age. Both anterior (**A**) and posterior (**B**) images of the whole-body bone scan revealed significantly increased MDP uptake in the left lower extremity. There was a hyperactive bone lesion with a central photopenic defect in the right mandible (arrow). In addition, there were increased radio-uptakes suspected to be degenerative lesions caused by scoliosis in the spine.

**Figure 3:**
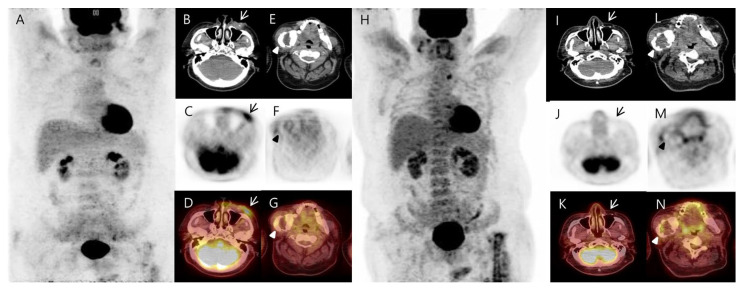
It was an unfamiliar finding for a diagnosis of osteomyelitis or heterotopic ossification. Therefore, we started taking a closer look at the patient’s medical history. He underwent surgery for basal cell carcinoma of the cheek just three years ago. At that time, a ^18^F-FDG PET/CT was performed to confirm the stage of the basal cell carcinoma on the face before surgery, and the scan showed a malignant lesion on the left cheek and a large hypermetabolic bone lesion in the right mandible. At the ^18^F-FDG PET/CT, the right mandible lesion was first considered a metastatic bone lesion, due to skin cancer. However, the degree of FDG metabolism in the lesion was not high enough to consider bone metastasis, and the pattern was unusual, so only the skin lesion was operated on; the right mandible lesion was followed up on. The left cheek lesion was confirmed as basal cell carcinoma upon histological examination. A ^18^F-FDG PET/CT was performed again six months after the skin cancer lesion surgery, and the skin cancer lesion seen on the left cheek was removed and was not visible. The right mandible lesion showed no significant change from before (Figure 3). Looking at it now, the right mandible lesion seen on the ^18^F-FDG PET/CT corresponded to the increased uptake area of the whole-body bone scan. Therefore, the right mandible lesion was considered a benign tumor, such as an odontogenic keratocyst. Reinterviewing the patient uncovered a history of mass removal from his right mandible when he was 15. The jaw mass removed at that time was an odontogenic keratocyst. After surgery, the jaw mass grew back, but he said he did not want to treat it. Maximum intensity projection (**A**) and axial images of the ^18^F-FDG PET/CT demonstrated (**A**) a hypermetabolic soft tissue mass, suggesting a malignant skin lesion in the left cheek (**B**–**D**, arrows). Additionally, a well-defined radiolucent mass lesion with sclerotic borders, cortical scalloping, and resorption of teeth roots was noted in the right mandible ((**E**)–(**G**), arrowheads). The lesion showed moderately increased FDG metabolism along its sclerotic border. On the ^18^F-FDG PET/CT images (**H**) obtained after six months of follow-up, the left cheek lesion was surgically removed and was not visible ((**I**)–(**K**), arrows). The right mandible lesion showed no significant interval change, compared to the previous images ((**L**)–(**N**), arrowheads). Unlike before, there was an abnormal increase in uptake in the left femur, but this was thought to be because the patient underwent surgery for osteomyelitis treatment between the previous images.

**Figure 4:**
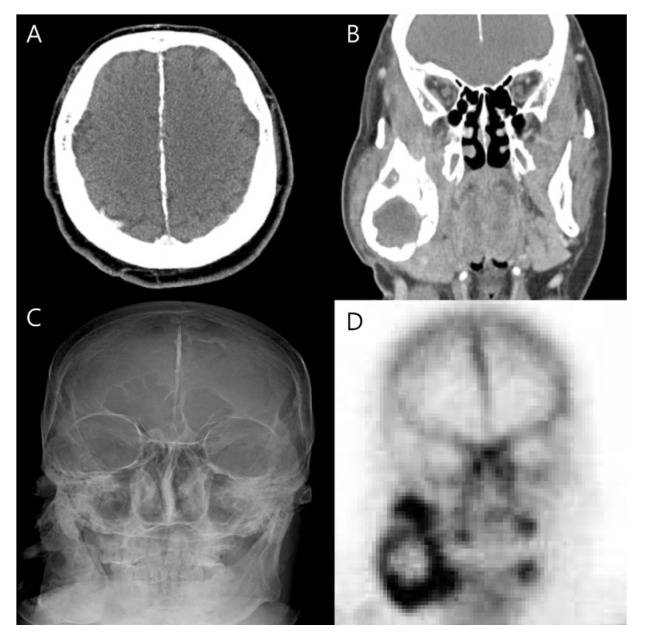
The diagnosis of Gorlin–Goltz syndrome was suspected at this time. As a result of reviewing extremity radiographic images and whole-body bone scans with this suspicion, we realized that this was due to skeletal anomalies and multiple ectopic calcifications. Investigation into the patient’s past radiographic images revealed additional findings consistent with Gorlin–Goltz syndrome. We found falx calcification, based on his old brain CT (Figure 4), and spinal scoliosis. Upon physical examination, he had macrocephaly and ocular hypertelorism. These findings, collectively, are diagnostic of Gorlin–Goltz syndrome. Finally, we formed the diagnosis based on the criteria for Gorlin–Goltz syndrome established by Evans et al. and modified by Kimonis et al. in 1997 [1,2]. According to them, diagnosis is based on the occurrence of at least two major or one major and two minor criteria (Table 1). Brain CT images (**A**,**B**), skull X-ray (**C**), and spot view of the whole-body bone scan (**D**). The spot view showed abnormal intense uptake over the expected area of the intercerebral falx. Additionally, the brain CT showed calcification within the falx cerebri. The skull X-ray showed lamellar calcifications of the falx. Although Gorlin–Goltz syndrome is very rare in routine practice, it usually represents a severe disease with involving multiple organ systems. It was first described by Gorlin and Goltz in 1960 as a triad of nevoid basal cell carcinomas, jaw cysts, and vertebral abnormalities [3]. The cause of the clinical manifestation is a mutation in the PTCH1 gene for the Patched receptor [4,5,6]. From a predictive point of view, early diagnosis with adequate therapy is critical. If a diagnosis is confirmed, lifelong dispensary care with interdisciplinary medical cooperation is necessary. Although the clinician who first meets the patient may be key for recognizing clinical suspicion of the syndrome, a multidisciplinary team is usually required for diagnosis, treatment, and follow-up. The described case illustrates several important points. Gorlin–Goltz syndrome is a disease in which multiple organ involvement can be seen and can resemble the metastatic process of basal cell carcinoma. A whole-body bone scan can be a useful imaging tool for evaluating other sites of skeletal involvement when the syndrome is suspected. In addition, we can discriminate between benign and metastatic bone tumors based on the degree of increased FDG metabolism on the ^18^F-FDG PET/CT in the mandibular mass. Our case highlights the multisystemic involvement of Gorlin–Goltz syndrome, based mainly on the skeletal findings. Practicing physicians, including imaging specialists, should be familiar with these findings to reach the diagnosis.

**Table 1:** Gorlin–Goltz syndrome diagnostic criteria.

Major Criteria	Minor Criteria
More than 2 basal cell carcinomas, one basal cell carcinoma at younger than 30 years of age, or more than 10 basal cell nevi	Congenital skeletal anomalies; fused, splayed, missing, or bifid ribs and wedged or fused vertebrae
Any odontogenic keratocyst or polyostotic bone cyst	Occipital–frontal circumference more than 97%
Three or more palmar or plantar pits	Hypertelorism
Falx cerebri calcification	Cleft lip and palate
Ectopic calcification	Cardiac or ovarian fibromas
Positive family history of Gorlin–Goltz syndrome	Medulloblastoma

## Data Availability

The data that support the findings of this study are available from the corresponding author M.C. upon reasonable request.
